# Fully Depleted Monolithic Active Microstrip Sensors: TCAD Simulation Study of an Innovative Design Concept

**DOI:** 10.3390/s21061990

**Published:** 2021-03-11

**Authors:** Lorenzo De Cilladi, Thomas Corradino, Gian-Franco Dalla Betta, Coralie Neubüser, Lucio Pancheri

**Affiliations:** 1 Dipartimento di Fisica, Università degli Studi di Torino, 10125 Torino, Italy; 2 Istituto Nazionale di Fisica Nucleare (INFN), Sezione di Torino, 10125 Torino, Italy; 3 Dipartimento di Ingegneria Industriale, Università degli Studi di Trento, 38123 Trento, Italy; thomas.corradino@unitn.it (T.C.); gianfranco.dallabetta@unitn.it (G.-F.D.B.); lucio.pancheri@unitn.it (L.P.); 4 Trento Institute for Fundamental Physics and Applications—Istituto Nazionale di Fisica Nucleare (TIFPA-INFN), 38123 Trento, Italy; coralie.neubueser@tifpa.infn.it

**Keywords:** particle detectors, silicon detectors, monolithic sensors, microstrip sensors, CMOS, TCAD simulations, fast timing

## Abstract

The paper presents the simulation studies of 10 μm pitch microstrips on a fully depleted monolithic active CMOS technology and describes their potential to provide a new and cost-effective solution for particle tracking and timing applications. The Fully Depleted Monolithic Active Microstrip Sensors (FD-MAMS) described in this work, which are developed within the framework of the ARCADIA project, are compliant with commercial CMOS fabrication processes. A set of Technology Computer-Aided Design (TCAD) parametric simulations was performed in the perspective of an upcoming engineering production run with the aim of designing FD-MAMS, studying their electrical characteristics, and optimizing the sensor layout for enhanced performance in terms of low capacitance, fast charge collection, and low-power operation. A fine pitch of 10 μm was chosen to provide high spatial resolution. This small pitch still allows readout electronics to be monolithically integrated in the inter-strip regions, enabling the segmentation of long strips and the implementation of distributed readout architectures. The effects of surface radiation damage expected for total ionizing doses of the order of 10 to 10${}^{5}$ krad were also modeled in the simulations. The results of the simulations exhibit promising performance in terms of timing and low power consumption and motivate R&D efforts to further develop FD-MAMS; the results will be experimentally verified through measurements on the test structures that will be available from mid-2021.

## 1. Introduction

Charged particle tracking and timing are fundamental tools for both physics research and for numerous applications. Although a number of detection techniques are available, silicon detectors have become largely employed due to their versatility and to the parallel strong developments of the semiconductor industry. Various types of silicon sensors have been developed to meet the specific requirements of different experiments and applications, such as high spatial resolution, fast charge collection, low power consumption, high radiation tolerance, and low cost per unit area.

Silicon detectors are divided in two categories, namely hybrid and monolithic detectors. The former are made of two separate silicon elements, the sensor and the chip, which are interconnected through external bump or wire bonding. While the sensor hosts the sensing volume only, the chip integrates the front-end readout electronics. On the contrary, monolithic sensors, which are emerging as a valid alternative to hybrid detectors, embed the front-end electronics in the same silicon substrate which hosts the sensing volume, with benefits in terms of material budget, production yield, and fabrication cost, as they are produced with commercial microelectronics processes [1,2,3].

Due to their characteristics, monolithic sensors have recently raised a wide interest in different research fields; studies, proposals, and developments have been made for applications in high energy physics (HEP) [4,5,6], X-ray imaging [7,8], medical particle imaging [9], and space experiments [10].

The state of the art includes three main types of monolithic sensors. The first type, called Depleted Field Effect Transistors (DEPFETs), is capable of low noise operation, thanks to low sensor input capacitance [11]. DEPFET detectors have been developed and used for HEP applications [12], for X-ray imaging in space [13] and for free electron laser experiments [14]. The main limitation of DEPFETs is the need to reset their internal gate which can be quickly saturated by the leakage [15], thus making this technology not suitable for environments with high levels of non-ionizing radiation.

A second approach consists in the SOI (Silicon-On-Insulator) monolithic sensors. SOI sensors embed a buried-oxide layer separating a thin low-resistivity silicon layer, which hosts the integrated readout circuitry, from a thicker high-resistivity substrate, which serves as the sensitive detection region [16,17]. This technology allows a low capacitance to be obtained [17]; however, SOI sensors suffer from back-gate effect and have a reduced radiation hardness, due to accumulation of positive holes charges in the buried oxide layer after irradiation [18]. Strategies have been found to overcome these limitations and to recover from the Total Ionizing Dose (TID) [19], but, as a consequence, the fabrication process of SOI sensors have become highly specialized and not compliant with standard microelectronics production processes. This results in increased cost per unit area, which is a critical issue for large-area detector applications.

A third category of monolithic sensors is represented by CMOS (Complementary Metal-Oxide Semiconductor) sensors [20]. CMOS sensors were already in use for light detection when they were first proposed for charged particle tracking at the beginning of the 2000s [21]. Over the last few years, important advancements in CMOS sensors allowed them to be employed in many applications, eventually leading to large scale productions for particle trackers at collider experiments. The STAR pixel detector, which took data at the Relativistic Heavy Ion Collider (RHIC) from 2014 to 2016, was the first large area monolithic pixel tracker ever built, for a total of 0.16 m${}^{2}$ [22]. These dimensions have been exceeded by the newly-constructed Inner Tracking System of the ALICE experiment at CERN, in which a total detector surface of about 10 m${}^{2}$ is covered by ALPIDE CMOS monolithic active (A monolithic sensors is called “active” if it integrates a signal amplifier inside each pixel or strip.) pixel sensors (MAPS) [4].

These achievements demonstrate the level of maturity and reliability that CMOS sensors have recently reached. However, there is still room for further improvements, especially in terms of charge collection speed and radiation hardness, and possibility to push previous limits in terms of low power density, high spatial resolution, and signal to noise ratio (SNR).

Pixel detectors are the first choice for small scale applications and for vertex trackers at collider experiments [2] as they have an intrinsic capability of providing a two-dimensional position information [23]. On the other hand, microstrip sensors [24] are widely used as particle detectors for space applications and are a competitive option for particle trackers due to their high spatial resolution, simpler readout and much lower power density (i.e., power consumption per unit area) compared to pixel detectors. Particle collider experiments have employed silicon hybrid strip sensors in the past and are still developing and assembling new trackers based on this technology, as in the case of the Phase-2 Upgrades of the CMS Outer Tracker [25] and of the ATLAS Strip Inner Tracker [26]. Recent space experiments equipped with silicon hybrid microstrip trackers include FERMI-LAT [27], DAMPE [28], PAMELA [29], and AMS-02 [30]. Strip-like sensors integrated in a monolithic technology have been proposed by combining the outputs of 55 μm × 55 μm [31] or 40 μm × 600 μm [32] pixels in each column or row of a pixel matrix.

Spatial resolution of 1.25–1.3 μm was achieved using hybrid silicon microstrip sensors with 25 μm pitch [33]. However, it has recently been demonstrated with fully depleted double-SOI monolithic pixel sensors that the 1 μm limit can be exceeded by semiconductor detectors [34]. The keys to a high spatial resolution with analogue readout are a fine microstrip pitch, a low sensor thickness to reduce Coulomb scattering and delta-ray emission, and an increased SNR, which can be achieved by reducing the leakage current and the sensor input capacitance to the readout electronics, but which is ultimately limited by the noise of the front-end electronics [24,33,35].

This paper presents the first investigation, design and simulation studies of CMOS Fully Depleted Monolithic Active Microstrip Sensors (FD-MAMS) with 10 μm pitch for charged particle detection. Properly optimized sensor layouts may allow sub-micron resolution, improved radiation hardness, and fast timing performance, thanks to full depletion [6,36] in a power-saving and cost-effective commercial technology. Moreover, a further advantage of monolithic microstrips is the potential complexity reduction of the detector assembly compared to hybrid microstrip detectors. In fact, since many readout functions can be monolithically integrated on the same chip which hosts the sensing volume, 1-by-1 strip bonding to the external readout electronics would not be needed anymore. Hence, we studied and designed the FD-MAMS within the framework of the INFN ARCADIA (Advanced Readout CMOS Architectures with Depleted Integrated sensor Arrays) project in order to provide an innovative solution for satellite-based space trackers and for particle detectors at future collider experiments.

The results of the Technology Computer-Aided Design (TCAD) simulations (The TCAD simulations were produced using the Synopsys^®^ Sentaurus (Version O-2018.06-SP2) software.) which allowed different MAMS design options to be compared in terms of sensor capacitance, reference voltage values, leakage current, and charge collection time and efficiency are presented; the effects of the inclusion of a silicon dioxide (SiO${}_{2}$) layer on top of the sensor and of surface radiation damage on the sensor operating parameters are explored; the study of charge sharing between groups of adjacent strips when particles with different Linear Energy Transfer (LET) traverse the sensor is reported. A selection of the MAMS presented in this paper is going to be implemented in test structures which were submitted in November 2020 for an engineering production run.

The paper is organized as follows: Section 2 presents the sensor concept for the ARCADIA fully depleted CMOS monolithic microstrip sensors and illustrates the set of parametric simulations that were performed for the sensor design; Section 3 describes and discusses the results of the simulations; Section 4 presents the conclusions, the future perspectives, and the planned tests for the ARCADIA monolithic microstrip sensors.

## 2. The ARCADIA Sensor Concept

The ARCADIA project and its precursor, SEED (Sensor with Embedded Electronics Development), designed an innovative sensor concept [37,38] based on a modified 110 nm CMOS process developed in collaboration with LFoundry and compatible with their standard 110 nm CMOS process. Up to 6 metal layers can be stacked on top of the sensor, for a total metal and insulator thickness of about 4–5 μm. The ARCADIA collaboration is developing a scalable event-driven readout architecture to cover detection surfaces of O(cm${}^{2}$) while maintaining ultra-low power consumption. The target for pixel sensors is 10 mW/cm${}^{2}$ to 20 mW/cm${}^{2}$ at rates in the order of 100 MHz, but, for less dense particle environments (e.g., in space applications), a dedicated low-power operation mode implements a cyclic pulling of the data packets from each section of the pixel matrix and disables most of the serializers and data transceivers, further reducing the total power consumption of the chip.

**Figure 1:**
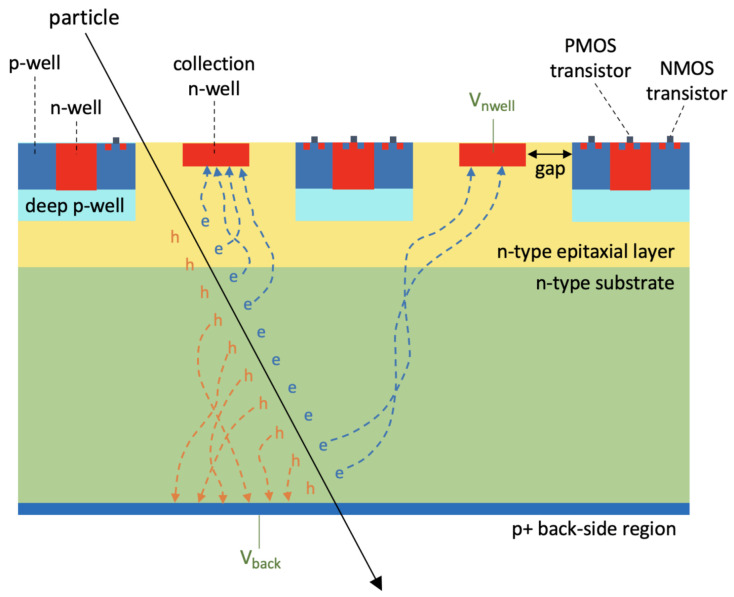
ARCADIA monolithic sensor concept. The dotted arrows indicate the drift path of electrons (e) and holes (h) generated by a particle crossing the sensor. The voltages V${}_{nwell}$ and V${}_{back}$ applied to the sensor contacts are shown in green. Not to scale.

In our project, an n-on-n sensor concept enabling full substrate depletion over tens or hundreds of microns and allowing full CMOS electronics to be implemented was employed. A simplified view of the sensor cross section is visible in Figure 1. The process allows to achieve sensor thicknesses from 50 to 400 μm. A high resistivity n-type substrate was used and constitutes the active volume. The sensing n-well node, located on top of the sensor, collects the electrons produced by ionization due to particles traversing the active detection volume.

N-doped and p-doped wells intended to host pMOSFETs and nMOSFETs, respectively, are shielded by a deep p-well, which allows the integration of full CMOS electronics and, hence, more complex digital functions, when necessary. In fact, the deep p-well prevents the n-wells hosting pMOSFETs from competing with the n-doped sensing node in the collection of the charge, thus avoiding loss of charge collection efficiency.

A p+ boron-doped region sits at the backside of the n-substrate, thus forming a pn-junction; when a negative bias voltage V${}_{back}$ is applied to the backside p+ contact, sensor depletion starts from the pn-junction at the bottom of the sensor and eventually extends to the whole sensor, if the backside voltage is sufficiently large. Since the high voltage needed for sensor depletion is applied at the backside, it is possible to maintain the voltage V${}_{nwell}$ applied to the front n-well electrode below 1 V and to use low-voltage integrated electronics (1.2 V transistors), which is more radiation-resistant and has lower noise. Full sensor depletion allows fast charge collection by drift (beneficial to enhance the timing performance), higher charge collection efficiency, deeper collection depth, and larger SNR; it also leads to improved radiation tolerance, as charge losses by trapping are reduced [5]. Since thicker sensors need higher backside bias voltage to reach full depletion, termination structures composed of multiple floating guard rings are used to avoid early breakdown at the edges of the pn-junction.

An additional n-type epitaxial layer, with lower resistivity than the substrate, is integrated between the n-type substrate and the deep p-wells. Its aim is to better control the potential barrier below the deep p-well, in order to delay the onset of the punch through current described in details in Section 2.2.2.

The feasibility of this sensor concept and approach to Fully Depleted monolithic CMOS sensors was proven in the framework of the SEED project [37,38], and the design activities described in this article have as a starting point the experimental and simulation work performed in that project. The main constraint on the design was to reduce to the minimum the modifications to the foundry’s standard fabrication process, in order to guarantee easy portability to different commercial fabrication processes. In the top side of the sensor, the only custom implant is the deep p-well. An additional constraint was the request to keep a low voltage on the sensor top surface to deal with possible embedded 1.2 V transistors.

The upcoming ARCADIA engineering run will include different design options of FD-CMOS monolithic sensors, both pixelated and strip-like. Large-area (1.3×1.3 cm${}^{2}$) pixel demonstrators with embedded CMOS electronics and pixel test structures (0.5×0.5 and 1.5×1.5 mm${}^{2}$) without integrated readout circuitry [39] are foreseen, with pitches ranging from 10 to 50 μm. The test structures will include, as well, the innovative MAMS and will allow a detailed characterization of these sensors. The 3D TCAD simulations performed to design the first FD-MAMS will be presented and discussed in the following.

### 2.1. TCAD Simulations

Three-dimensional TCAD simulations were employed as a tool to design the sensors and study their performance. The use of 3D simulations is necessary to have a more realistic domain and results which are more accurate and less affected by boundary conditions. Furthermore, we were also interested in studying the charge collection dynamics after a particle crosses the sensor, and this is more straightforward with 3D simulations. A fine pitch of 10 μm was chosen for the microstrips in order to explore the characteristics and performance of a sensor layout which pushes the requirements on both spatial and timing resolution. Different sensor thicknesses foreseen for the production runs were simulated. Variations in the sensor layout and operating parameters were tested to study and optimize the sensor response. The simulated sensor options take into account the limitations imposed by the foundry’s fabrication process, especially for the n-well and p-well sizes. The strip simulations investigated sensor layouts which pushed the design to the limits of the process requirements.

**Figure 2:**
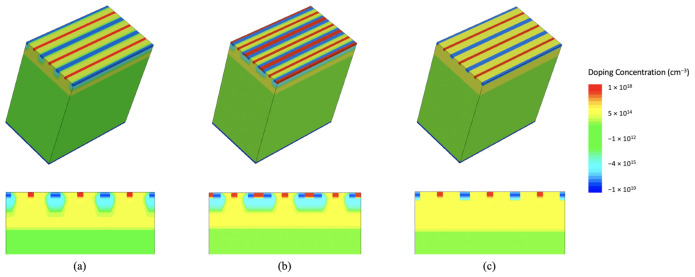
Example Technology Computer-Aided Design (TCAD) 3D sensor domains for ARCADIA microstrips (top row) and corresponding cross sections (bottom row). (**a**) Standard simulation domain for sensors with the deep p-well. (**b**) Addition of n-wells above the deep p-wells. (**c**) Simulated ARCADIA microstrips without deep p-wells.

All the TCAD simulations were performed at a temperature of 300 K. A standard simulation domain including three 50 μm long, 50 μm thick, 10 μm pitch microstrips is shown as an example in Figure 2a. The n-doped substrate is shown in green, the epitaxial layer in yellow, the microstrip sensing n-wells in red, the p-wells in blue, and the less doped deep p-well in light blue. The default value for V${}_{nwell}$ is 0.8 V. The p-wells, instead, are kept at a voltage V${}_{pwell}=0$ V.

One of the simulated sensor options has been specifically designed to allow for CMOS digital library cells to be integrated along the strips and is shown in Figure 2b. This sensor variant would allow the deployment of complex CMOS digital functions along the strip for distributed signal processing. We observed that the n-wells dedicated to the implementation of PMOS transistors and shielded by the deep p-well do not significantly influence the electrical characteristics of the detector in the TCAD simulation results. Therefore, we did not include them in the simulations.

The deep p-well can be removed in the test structures that will be used to characterize the sensor (see Figure 2c), and the necessary CMOS front-end electronics can be deployed at the end of the strips in the chip periphery. Sensors without the deep p-well were simulated, as well.

Different n-well, p-well, and deep p-well sizes were considered to find the optimal layout in terms of sensor performance. Simulations were also employed to predict the effects that possible production uncertainties can have on the sensor operating parameters and electrical characteristics. For instance, the thickness and resistivity of the epitaxial layer may vary within a confidence range around their typical specified value (see Appendix A). Three-dimensional simulations for the different cases were run and compared. Some simulation parameters were fine-tuned using characterization results from a previous set of test structures, produced in the framework of the SEED project [37].

### 2.2. Electrical and Transient Simulation

In this section, the simulations performed to extract the sensor electrical characteristics and to study the charge collection dynamics are briefly illustrated. Shared definitions and conventions on simulation setups and operating parameters were agreed for all the ARCADIA sensor simulations and are also described by Neubüser et al. [39]. The strip length in the upcoming production run will be 1.2 cm. However, MAMS with lengths of 50 μm were simulated in order to run the TCAD simulations in a reasonable computational time. The results were then scaled to the desired length.

#### 2.2.1. Depletion Voltage

**Figure 3:**
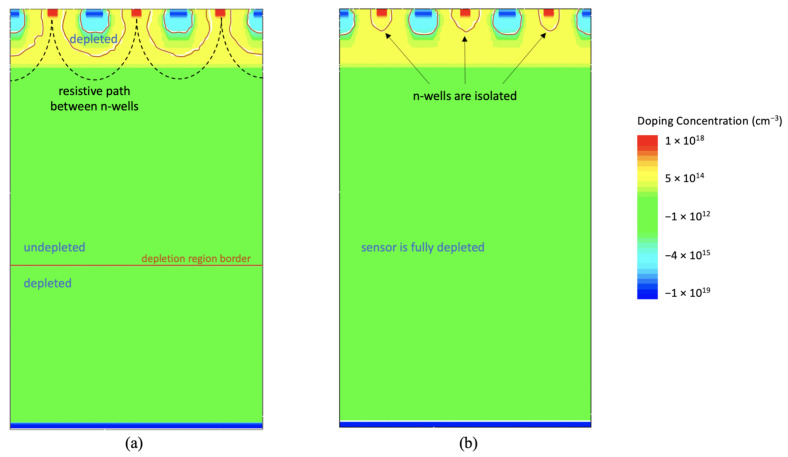
Depletion process in ARCADIA microstrips. (**a**): cross section of a sensor before full depletion is reached. (**b**): cross section of a fully depleted Monolithic Active Microstrip Sensors (MAMS). The orange lines indicate the edge of the depletion region.

Sensor depletion starts at the backside, where the pn-junction between the n-type substrate and the p+ contact is located. If no negative bias voltage is applied to the backside contact, the sensor is not fully depleted, and the collection n-wells are not isolated. This means that a resistive path exists between the n-type sensing nodes (see Figure 3a). Therefore, if a voltage difference is applied between two adjacent n-wells, a current will flow between them.

As the negative voltage applied to the backside contact increases, the space charge region enlarges through the high resistivity substrate, eventually merging with the depletion volume which surrounds the pn-junctions formed between the n-type substrate or epitaxial layer and the deep p-wells. At this point, the sensor is fully depleted, the resistive path between the sensing nodes is closed, and the collection n-wells are isolated; this is shown in Figure 3b. In this condition, no current (except for the leakage current) will flow among adjacent n-wells even when different voltages are applied to them.

**Figure 4:**
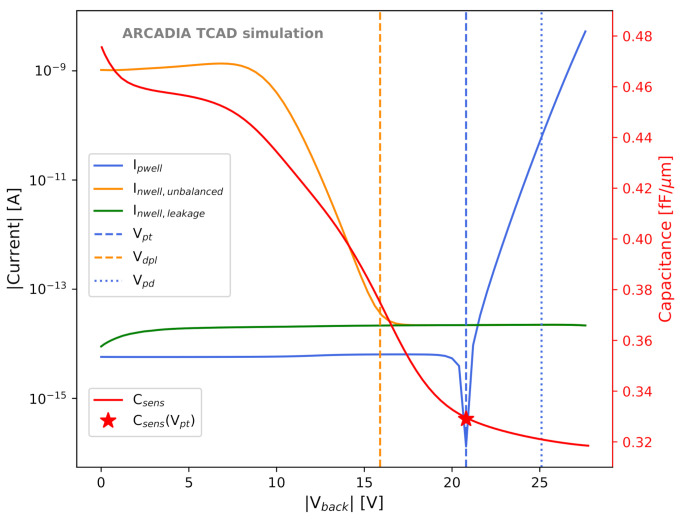
Example characteristic IV and CV curves extracted from TCAD simulations of ARCADIA monolithic sensors. The red vertical axis refer to the sensor capacitance ($C_{sens}$) CV curve.

This behavior can be observed in the orange example IV (current-voltage) curve in Figure 4 ($I_{nwell,unbalanced}$). The simulated domain shown in Figure 2a was used. In this simulation, a voltage unbalance of 10 mV was applied between adjacent strips: the first n-well was biased at 0.79 V, the central one at 0.8 V, and the third one at 0.81 V. The curve shows the current measured at the sensing node of the central strip as a function of $|V_{back}|$. A current of about 1 nA is measured at $V_{back}=0$ V. As the backside voltages increases and the space charge region enlarges, the current starts decreasing, and eventually reaches a plateau at a current of about $10^{-5}$ nA. This baseline corresponds to the leakage current (green IV curve, $I_{nwell,leakage}$). The backside voltage at which the single microstrips become isolated and the plateau is reached is the sensor depletion voltage $V_{dpl}$; this voltage is evaluated as the intersection point between the exponential decay fitting of the IV curve decreasing segment and the baseline.

**Figure 5:**
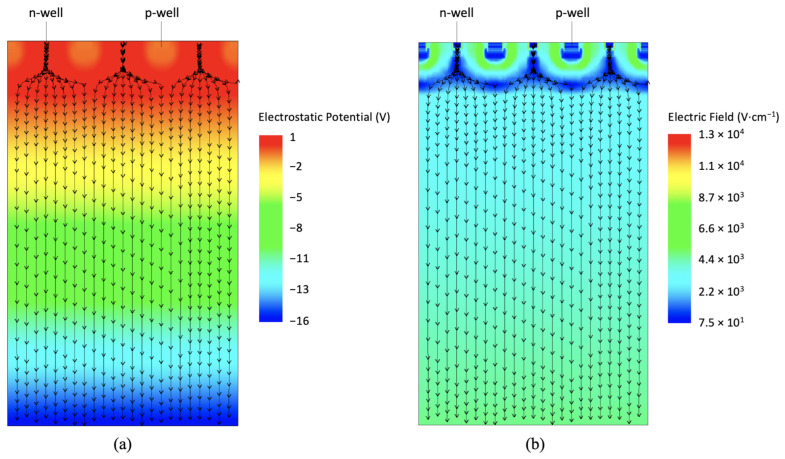
Electrostatic potential map (**a**) and electric field map (**b**) for a group of three ARCADIA microstrip sensors at $V_{back}=V_{dpl}$. The electric field lines are plotted on top of both maps.

Figure 5 shows the simulated electrostatic potential and electric field maps at $V_{back}=V_{dpl}$ in a cross section of a 3-strip domain with all the n-wells at $V_{nwell}=0.8$ V. Electric field lines are plotted on top of both the electrostatic potential and the electric field maps.

#### 2.2.2. Punch-Through

If $V_{back}$ exceeds a certain value, a hole current flowing between the shallow p-doped backside region and the (deep) p-well exponentially increases. This condition is known as punch-through, and the hole current is the punch-through current [40]. We define the voltage corresponding to the onset of the punch-through as $V_{pt}$. The onset of the punch-through currents can be observed from the blue IV curve in Figure 4 ($I_{pwell}$), which shows the absolute value of the current measured at the top p-well contacts as a function of $|V_{back}|$. The dip in the curve, corresponding to the point of sign inversion of the current, was defined as $V_{pt}$. The simulation domain includes three 50 μm long, 50 μm thick, 10 μm pitch microstrips. In this case, the n-wells are all biased at $V_{nwell}=0.8$ V, which is the default value.

Sensor operation in low punch-through regime can be tolerated, whereas a punch-through current density exceeding a value of the order of 100 μA/cm${}^{2}$ ought to be avoided, as it determines a substantial increase in the power consumption of the whole detector. For this reason, we chose $V_{back}$ = $V_{pt}$ as a safe reference sensor operating voltage; this is the operating point for all the results shown in the following, if not stated differently. The sensor power density can be defined as $pd=\frac{V_{back}\times \left(I_{pwell}+I_{nwell}\right)}{A}$, where $I_{nwell}$ and $I_{pwell}$ are the currents flowing at the sensing node and at the top p-well contacts, respectively, and *A* is the top surface area of the simulated microstrip domain. In order to quantify the maximum acceptable backside bias voltage that limits the absorbed power density, the value $V_{pd}$ at which pd=0.1 mW/cm${}^{2}$ was extracted from the simulated IV curves (see Figure 4).

**Figure 6:**
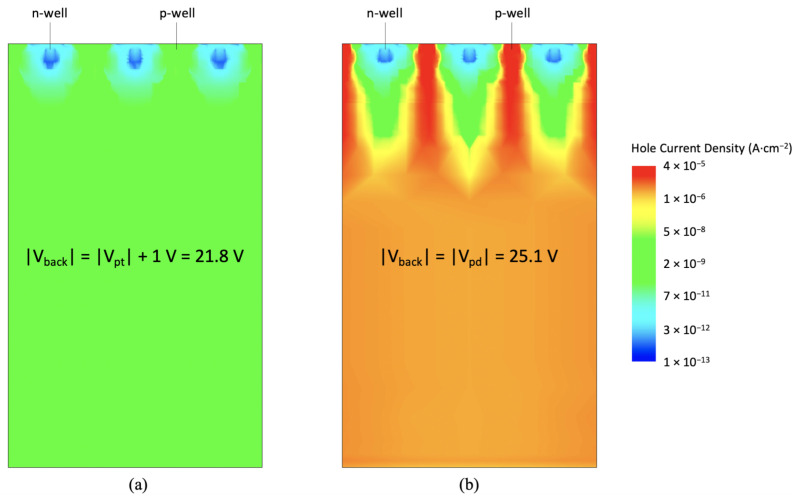
Hole current density in a simulated sensor domain including three microstrips in punch-through condition at two different $V_{back}$.

Figure 6 shows the hole current density at two different $|V_{back}|$ > $|V_{pt}|$ in the simulation domain used to extract the $I_{pwell}$ curve of Figure 4. In Figure 6a, a backside voltage exceeding $V_{pt}$ by 1 V was chosen, while, in Figure 6b, $V_{back}$ was set to $V_{pd}$. An increase in the hole current density of several orders of magnitude can be observed below the deep p-wells and in the substrate.

Care had to be taken to ensure that $|V_{dpl}|$ < $|V_{pt}|$ in the designed sensors. In this way, full depletion is reached before the onset of the punch-through. Moreover, the voltage operating range between $V_{dpl}$ and $V_{pt}$, defined as $\Delta V_{op}=\left|V_{pt}-V_{dpl}\right|$, should be large enough to ensure safe operation in full depletion before the onset of the punch-through even if deviations from the simulated design occur in the sensor fabrication process.

#### 2.2.3. Leakage Current

The same sensor domain and n-well voltage configuration used for the extraction of $V_{pt}$ was also used to evaluate the sensor leakage current $I_{leak}$. The leakage current is defined as the current flowing at the collection nodes in full depletion and in absence of external stimuli, such as particles or radiation. The leakage current as a function of the backside bias voltage is shown in Figure 4 as a green curve ($I_{leak}$). In the example shown in Figure 4, a value of 10 fA was extracted for $I_{leak}$ at $V_{back}=V_{pt}$.

#### 2.2.4. Sensor Capacitance

The sensor CV (capacitance-voltage) curve was simulated through AC simulations with a frequency of 10 kHz using the same sensor domain employed for $V_{pt}$ and $I_{leak}$ evaluation, with $V_{nwell}=0.8$ V. The major contribution to the sensor capacitance $C_{sens}$, which is the input capacitance seen by the DC-coupled front-end electronics, originates from the lateral capacitance between the collection n-well and the surrounding p-wells. Thus, it is important to minimize this contribution by a careful selection of the distance between the edge of the collection n-well and the p-wells; we call this distance “gap” (see Figure 1). An example CV curve is shown in red in Figure 4, with the capacitance per unit length considered. In the example of Figure 4, a value of about 0.33 fF/μm was obtained at $V_{back}=V_{pt}$.

It has to be mentioned that in these sensors the depletion voltage does not necessarily correspond to the voltage of minimum capacitance. The reason for this is the presence of the epitaxial layer, which is located far from the backside pn-junction and has a lower resistivity than the substrate. Therefore, the depletion of the epitaxial layer begins after the depletion of the substrate and progresses more slowly with voltage. Full depletion of the whole sensor, including the epitaxial layer, and minimum capacitance are only reached at $|V_{back}\left|>\right|V_{dpl}|$. From this point, both capacitance and leakage current values will be intended at $V_{back}=V_{pt}$.

A central focus of the design was the minimization of the sensor capacitance. In fact, low input capacitance to the DC-coupled CMOS readout electronics allows for low-noise readout, low analog power [5], and, in particular, SNR maximization. Large input capacitance worsens the noise levels and the speed of the front-end electronics [6].

#### 2.2.5. Surface Radiation Damage

In the set of simulations performed to study the properties of MAMS, a silicon dioxide (SiO${}_{2}$) layer was added on the top-side of the sensor. In addition to this, surface damage was modeled to evaluate the effects of Total Ionizing Dose (TID) on the sensor electrical properties.

**Figure 7:**
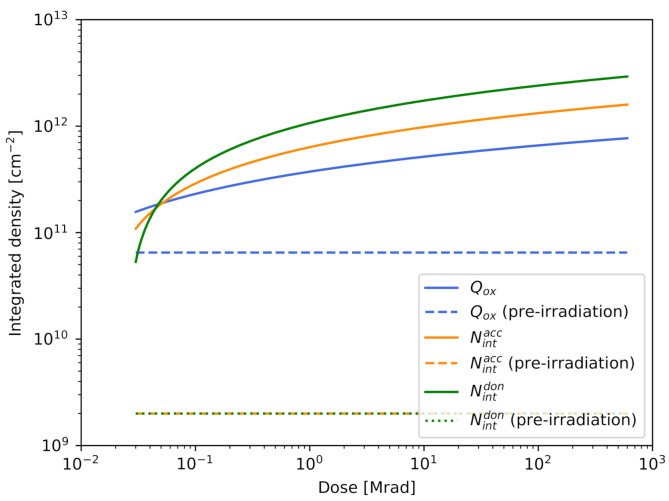
Dependence of the oxide charge density $Q_{ox}$, acceptor integrated interface trap state density $N_{int}^{acc}$, and donor integrated interface trap state density $N_{int}^{don}$ on the dose for the surface radiation damage model described by Passeri et al. [41]. Pre-irradiation values are shown as horizontal dotted lines.

The impact of surface radiation damage was modeled following the AIDA-2020-D7.4 report [41]. The model introduces fixed positive oxide charges and band-gap acceptor/donor defect levels (trap states) at the Si-SiO${}_{2}$ interface. The concentrations of oxide charges and defect levels start from a fixed value before irradiation (i.e., with the only inclusion of the SiO${}_{2}$ surface layer, at dose=0) and increase with the dose provided to the sensors. The dependence of the oxide charge density $Q_{ox}$ (charges × cm${}^{-2}$), of the acceptor integrated interface trap state density $N_{int}^{acc}$ (cm${}^{-2}$), and of the donor integrated interface trap state density $N_{int}^{don}$ (cm${}^{-2}$) on the dose is shown in Figure 7. Pre-irradiation values, shown as dotted horizontal lines in Figure 7, are $Q_{ox}=6.5\times 10^{10}$ charges × cm${}^{-2}$, $N_{int}^{acc}=2.0\times 10^{9}$ cm${}^{-2}$, and $N_{int}^{don}=2.0\times 10^{9}$ cm${}^{-2}$.

In the simulations, the effects of the inclusion of the SiO${}_{2}$ layer and of the radiation damage on the leakage current, sensor capacitance, depletion voltage, and punch-through voltage were investigated and will be discussed in Section 3.

#### 2.2.6. Transient Simulations

TCAD transient simulations were run to study the sensor charge collection process in response to particles traversing the simulated microstrip domain. These simulations also let us identify the most relevant layout parameters to be optimized for improving the sensor performance in terms of fast and uniform charge collection irrespective of the particle incidence position. The transient simulations employ the Synopsys^®^ Sentaurus TCAD HeavyIon model, described in Sentaurus Device User Guide [42]. The HeavyIon model gives an analytical description of the amount of charge generated within a 3D cylindrical distribution along the incident particle track. Two main parameters have to be passed to the HeavyIon model: the Linear Energy Transfer (LET), defined as the average deposited charge per unit length, and the transverse size of the charge deposition volume generated around the particle trajectory. We chose the charge transverse distribution profile to be gaussian around the particle track.

**Figure 8:**
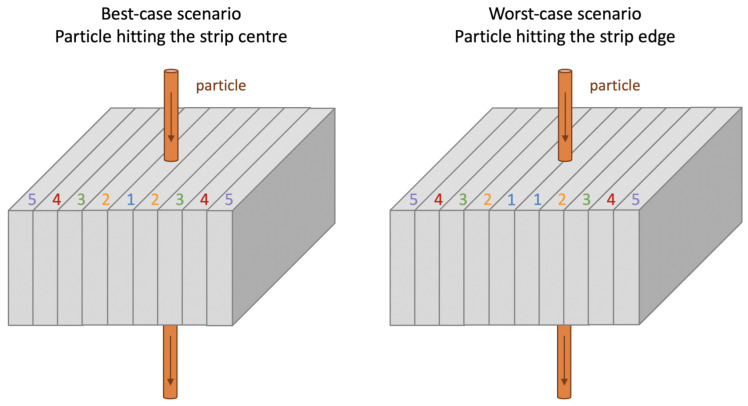
Best-case and worst-case scenarios considered in the TCAD transient simulations. The microstrips are represented as adjacent grey blocks, and the particle traversing the domain is shown as an orange cylinder. The nomenclature used to identify the microstrips (from 1 to 5) is illustrated.

Two extreme cases in terms of particle impact position were studied to evaluate the uniformity of charge collection time and charge collection efficiency. Particle trajectories perpendicular to the sensor surface were considered. In the best-case scenario, the particle impact point corresponds to the centre of a microstrip, which is the centre of a collection n-well. On the contrary, in the worst-case scenario, the particle traverses the sensor at the edge between two adjacent microstrips, i.e., in the middle of a p-well. In Figure 8, the two cases and the corresponding numbering of the strips are illustrated. This conventional strip nomenclature will be used in the following when referring to transient simulations.

**Figure 9:**
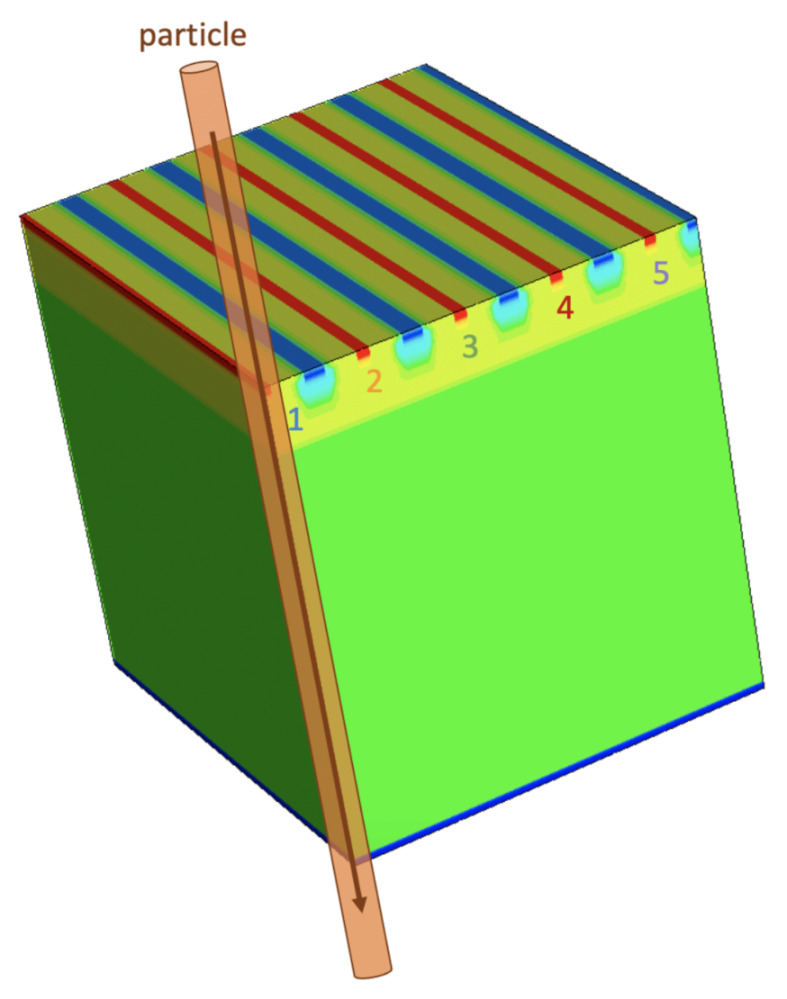
Example reduced TCAD domain used in transient simulations (best-case scenario). The microstrips are labeled following the nomenclature illustrated in Figure 8. A crossing particle is represented as an orange cylinder hitting the corner of the simulated reduced domain.

In order to save computational time, a reduced TCAD simulation domain that employs the symmetries was used. This reduced domain corresponds to a quarter of the full domain, with the particle incident in the corner of the domain instead of in the centre. An example for the best-case scenario is shown in Figure 9. The collected charge and current signals were then scaled to reproduce the full domain case, which includes nine or ten 100 μm long microstrips in the best-case and worst-case scenario, respectively (Figure 8). These numbers and size of strips guarantee that the amount of deposited charge reaching the borders of the simulation domain is negligible. The correctness of this strategy was verified and confirmed by comparing the results of a simulation with a quarter domain and of a simulation with full domain.

**Figure 10:**
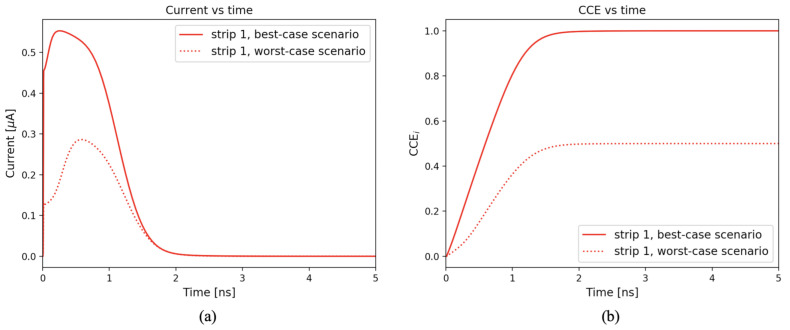
Simulated current signals (**a**) and corresponding charge collection efficiency CCE${}_{i}$ (**b**) in the best-case and worst-case scenarios for strip number 1 in an example 50 μm thick microstrip domain. A particle track with an Linear Energy Transfer (LET) of $1.28\times 10^{-5}$ pC/μm was simulated.

An example of current signals $I_{nwell}\left(t\right)$ measured at the microstrip sensing nodes when a particle crosses the microstrip domain is shown in Figure 10a. We defined as charge collection efficiency for the i-th strip (CCE${}_{i}$) the integral of the current signal $I_{nwell,i}\left(t\right)$ extracted from the i-th strip and normalized at the total charge $Q_{tot}$ deposited in the sensor by the particle, according to the formula:

(1) $$CCE_{i}\left(t\right)=\frac{\int_{0}^{t}I_{nwell,i}\left(t^{\prime }\right)\;dt^{\prime }}{Q_{tot}}=\frac{\int_{0}^{t}I_{nwell,i}\left(t^{\prime }\right)\;dt^{\prime }}{LET\times d_{Si}},$$

where $d_{Si}$ is the sensor thickness. The total charge collection efficiency CCE for the whole simulated domain is defined as:

(2) $$CCE\left(t\right)=\sum_{i=1}^{N_{strips}}CCE_{i}\left(t\right),$$

where $N_{strips}$ is the total number of strips in the simulated domain. The total CCE at the end of the charge collection process (i.e., at $t=t_{end}=30$ ns, which was observed to be enough for complete charge collection) has to be equal to 100% in the absence of recombination:

(3) $$CCE(t=t_{max})=100\%.$$

The CCE${}_{i}$ as a function of time is shown in Figure 10b, for strip number 1. The times needed for collecting the 95% and 99% of the total deposited charge were evaluated and referred to as $t_{95}$ and $t_{99}$, respectively. These values were compared for different design options and used to select the fastest sensor layouts.

The spatial mesh of the transient simulations was forced to be finer around the particle trajectory to more accurately simulate the charge deposition and the drift of electrons and holes from their generation points along the particle track towards the electrodes. Additionally, the time step of the transient simulations was fine tuned to guarantee the necessary accuracy while keeping the computational time requirement economical. We observed that these adjustments prevented the simulations from giving unphysical results.

### 2.3. Determination of the LET for Heavy Nuclei

**Figure 11:**
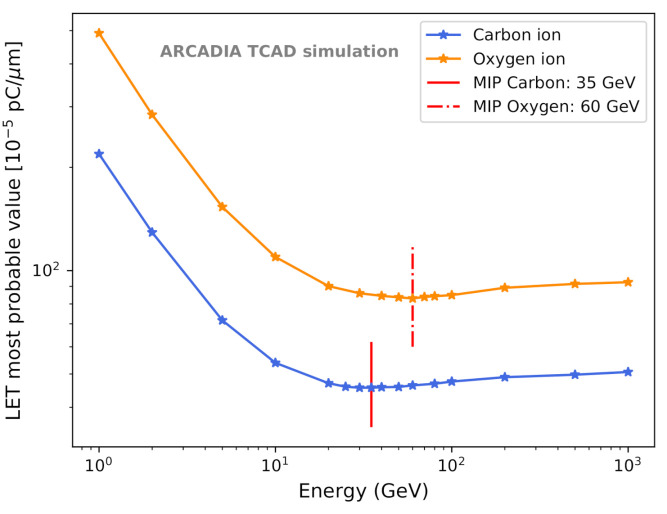
Dependence of the LET on the energy of carbon ions (C${}^{12+}$, blue) and oxygen ions (O${}^{16+}$, orange) incident on 50 μm thick silicon. The LET values were evaluated through Geant4 simulations. The red vertical lines indicate the minimum ionization energies for the two particle species.

Since MAMS are an interesting candidate for tracking detectors in space applications, the charge collection was studied not only for minimum ionizing particles (MIPs) but also for heavy nuclei of interest for in-orbit astroparticle experiments. The LET values of carbon and oxygen ions were studied in Geant4 (version 10.6 patch 01) simulations. The simulation setup consists of a 50 μm thick silicon layer immersed in air, with a transverse size of 1 × 1 cm${}^{2}$. The particle gun was positioned 15 cm in front of the centre of the silicon layer. The *G4EmPenelopePhysics* physics list was used to model the electromagnetic processes and the necessary precision on the energy deposited within the silicon was achieved with a maximum step size of 1 μm [43]. This setup has been validated with simulations of 300 MeV muons, where the known LET for MIPs of about 80 electron-hole (e-h) pairs per μm [44] (or $1.28\times 10^{-5}$ pC per μm) in silicon could be reproduced. The LETs for carbon (C${}^{12+}$) and oxygen (O${}^{16+}$) ions at their minimum ionization were computed from their most probable energy loss (i.e., the most probable value of the straggling or Landau functions [45,46]). Figure 11 shows the LET as a function of the particle energy obtained for C and O ions traversing 50 μm of silicon. The energies $E_{min}$ at which C and O ions are at the minimum of ionization were found to be 35 GeV and 60 GeV, respectively. The corresponding LETs are $45.6\times 10^{-5}$ pC/μm and $83.0\times 10^{-5}$ pC/μm, which results in 36 and 65 times the MIP value. This is consistent with the expected scaling from the Bethe-Bloch formula.

We were especially interested in studying the charge sharing among the microstrips surrounding the particle impact point and the charge collection time at different LETs. This will be reported and discussed in Section 3.

## 3. Results and Discussions

In this section, the results of the TCAD simulations will be presented in connection with the design objectives. As mentioned in Section 1, the main targets of the FD-MAMS design were the following.

1. To enhance the spatial resolution. The microstrip pitch of 10 μm means an intrinsic spatial resolution of $\frac{pitch}{\sqrt{12}}≃2.9$ μm with a digital readout, which can be improved, thanks to charge sharing and with an analog readout.
2. To minimize the sensor capacitance $C_{sens}$ at $V_{back}=V_{pt}$.
3. To obtain fast and uniform charge collection, irrespective of the particle incidence position. This will enhance the sensor timing capabilities and will reduce the dead-time between successive particle detections.

For reasons of space available for MAMS in the first ARCADIA engineering run, only a few sensor layout options could be included. Hence, a set of parametric simulations was needed to identify the best performing sensor layouts. The deep p-well, when present, was kept the same size as the p-well. The expression “p-well and deep p-well” will be contracted and referred to as “(deep) p-well”. In the legends of the figures, the abbreviation “dpw” will be used for deep p-well.

### 3.1. SiO${}_{2}$ Layer and Surface Damage

**Figure 12:**
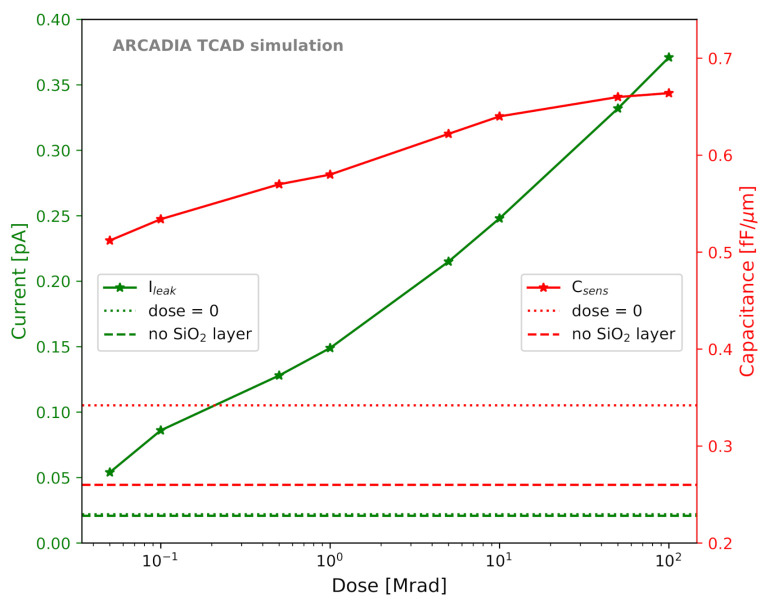
Leakage current $I_{leak}$ (green) and sensor capacitance $C_{sens}$ (red) as a function of the total ionizing dose for a 50 μm thick microstrip sensor. The values obtained in simulations with and without the SiO${}_{2}$ layer in the absence of irradiation are shown as horizontal lines and referred to as “dose = 0” and “no SiO${}_{2}$ layer”, respectively.

**Figure 13:**
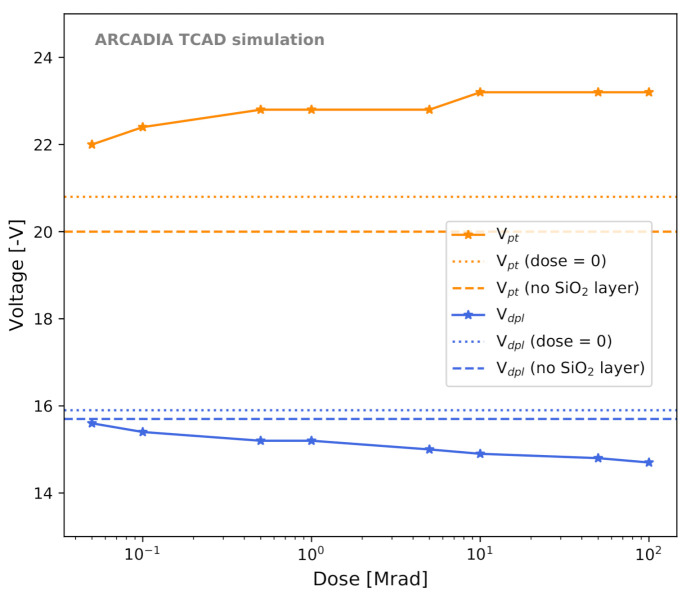
Depletion voltage $V_{dpl}$ (blue) and punch-through voltage $V_{pt}$ (orange) as a function of the total ionizing dose for a 50 μm thick microstrip sensor. The values obtained in simulations with and without the silicon dioxide layer in the absence of irradiation are shown as horizontal lines.

A first group of TCAD simulation studies was aimed at investigating the effects of the SiO${}_{2}$ layer and of surface TID damage on the FD-MAMS characteristics. The model we employed was presented in Section 2.2.5. As can be seen from Figure 12, for one of the selected 50 μm thick microstrip layouts, the inclusion of the SiO${}_{2}$ layer with a minimum concentration of traps and oxide charges (dose=0) determines a small increase of about 5% in the leakage current $I_{leak}$ from 20.8 fA to 22.0 fA. The sensor capacitance $C_{sens}$ is strongly affected by the inclusion of the SiO${}_{2}$ layer, as it increases by 31% from 0.26 fF/μm to 0.34 fF/μm. Both $I_{leak}$ and $C_{sens}$ are found to rise with increasing dose. The minimum dose that we considered is 50 krad, as the model is not validated for lower doses [41]. Figure 13, instead, shows the effect of the SiO${}_{2}$ layer and of the TID on $V_{dpl}$ and on $V_{pt}$. The effect of the dose on these two values is smaller than in the case of $I_{leak}$ and $C_{sens}$. Furthermore, $V_{dpl}$ and $V_{pt}$ are influenced by the dose in opposite directions, which results in a slight increase in the operating range ΔV${}_{op}$ with increasing dose.

**Figure 14:**
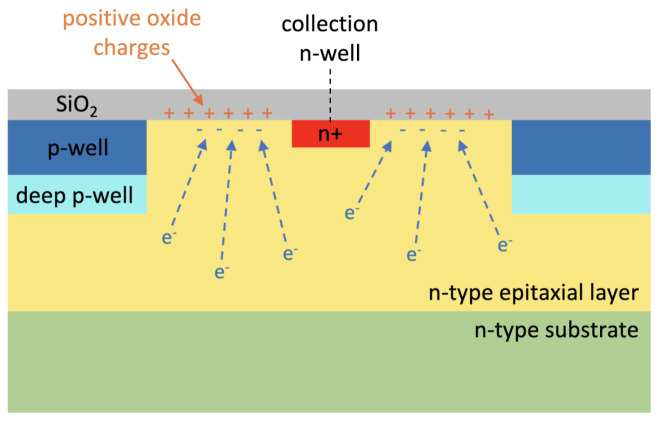
Schematic illustration of the electron accumulation in the gap between the collection n-well and the surrounding p-wells due to the positive oxide charges introduced at the Si-silicon dioxide (SiO${}_{2}$) interface.

**Figure 15:**
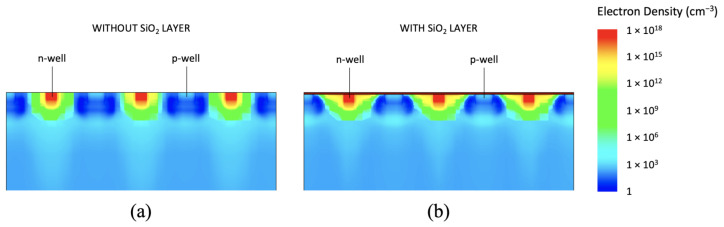
Electron density in an example microstrip simulation domain without (**a**) and with (**b**) the SiO${}_{2}$ layer on top of the sensors.

The reason for the capacitance increase even after the simple inclusion of the SiO${}_{2}$ layer was found to be the introduction of positive oxide charges at the Si-SiO${}_{2}$ interface [39]. In fact, the model that we adopted foresees a significant positive oxide charge concentration $Q_{ox}$ = 6.5 × $10^{10}$ charges/cm${}^{-2}$ already at dose = 0. The positive oxide charges attract free electrons from the n-type silicon epitaxial layer towards the Si-SiO${}_{2}$ interface in the gap. This determines an increase in the electron concentration in the gap, as illustrated in Figure 14 and Figure 15, which behaves as an extension of the collection n-well.

### 3.2. Capacitance Minimization

**Figure 16:**
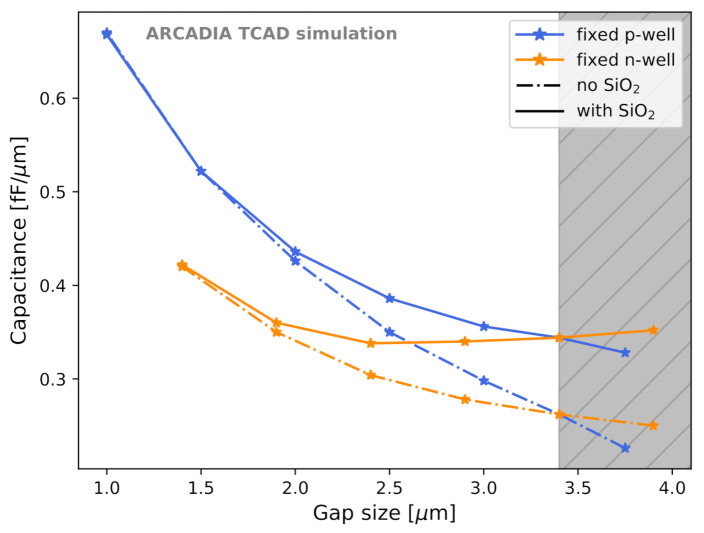
$C_{sens}$ as a function of the gap size for different sensor layout configuration. The vertical grey band is the forbidden region due to fabrication constraints; its leftmost limit is still permitted.

The sizes of both the collection n-well and of the gap were found to contribute to $C_{sens}$ and were adjusted to reach minimum capacitance. The SiO${}_{2}$ layer influences $C_{sens}$ in different ways for different gap sizes. Thus, the sensor capacitance was evaluated with and without the SiO${}_{2}$ layer to quantify its effect. Figure 16 shows the trend of $C_{sens}$ as a function of the gap size for 50 μm thick microstrips. The different sensor thicknesses considered (50, 100, and 300 μm) were found not to influence the sensor capacitance. Both the case with fixed minimum-size n-well and variable (deep) p-well (blue curves) and the case with fixed minimum-size (deep) p-well and variable n-well (orange curve) were studied. The dash-dotted lines refer to simulations without the surface SiO${}_{2}$ layer, whereas solid lines to the case with SiO${}_{2}$ layer included with minimal oxide charge and trap concentration.

The reason for which smaller gaps with fixed n-wells could not be investigated is referred to as *channel choking*, a condition that inhibits sensor operation; this condition is explained in Section 3.3. The vertical grey band in Figure 16 and in the following ones corresponds to the forbidden region due to the constraints on n-well and (deep) p-well minimum sizes imposed by the fabrication process. The leftmost limit of the grey band is still permitted.

Variations of n-well and of (deep) p-well size do not lead to the same $C_{sens}$ for the same gap size. A fixed n-well size with SiO${}_{2}$ layer included shows a trend that is not monotonic, but has a minimum at slightly less than 0.34 fF/μm. This effect is caused by the electron accumulation in the gap at the Si-SiO${}_{2}$ interface. However, the difference in $C_{sens}$ between the minimum-capacitance option and the sensor layout at the edge of the forbidden region is lower than 2%. There was, as expected, no benefit from having large n-wells. The sensor capacitance increases with the n-well size, as can be seen from the blue curve in Figure 16. Therefore, the best layout for minimum $C_{sens}$ has the smallest possible n-well size and sufficiently small (deep) p-well.

**Figure 17:**
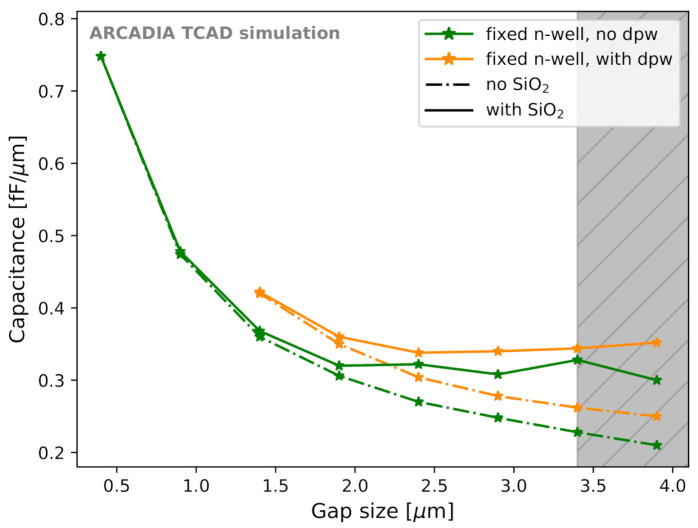
Sensor capacitance $C_{sens}$ as a function of the gap size for different sensor layout configurations with and without the deep p-well.

After fixing the n-well to the minimum possible size, we compared the sensor capacitance for layouts with deep p-well (Figure 17, orange curve) and without deep p-well (Figure 17, green curve). It was observed that removing the deep p-well helps in reducing the sensor capacitance.

### 3.3. Reference and Operating Voltages

**Figure 18:**
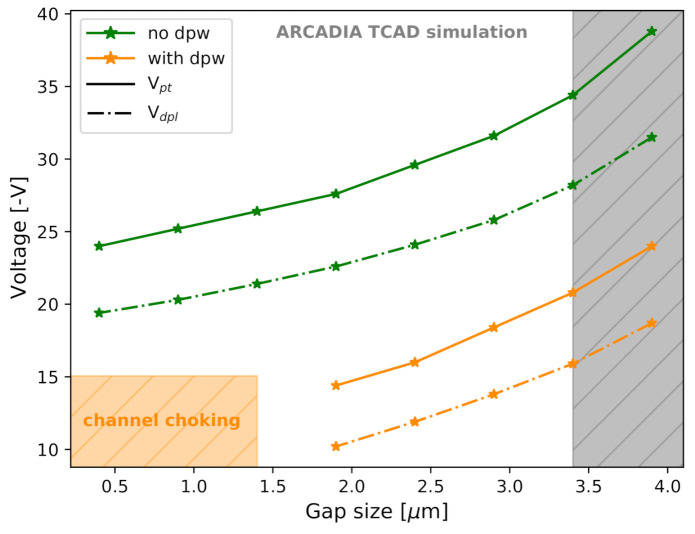
Sensor depletion voltage $V_{dpl}$ and punch-through voltage $V_{pt}$ as a function of the gap size for different sensor layout configurations. The orange region indicates the forbidden region due to the observed *channel choking*.

The influence of the n-well size on the operating voltages was negligible compared to the effect of the (deep) p-well size. Figure 18 presents the effect of the (deep) p-well size effect on $V_{dpl}$ and on $V_{pt}$ for 50 μm thick sensors with minimum-sized n-well. Both the cases with (orange curves) and without deep p-well (green curves) were considered. The voltage values are reported for the case of dose = 0.

In all the layouts considered in Figure 18, the onset of the punch through happens at voltages sufficiently larger than the depletion voltage. Outside of the forbidden region (grey band), the operating range $\Delta V_{op}$ is always between 4.2 V and 6.2 V, or between the 23% and the 41% of $V_{dpl}$. This is sufficient for safe sensor operation, even in the hypothesis of possible doping inhomogeneities among adjacent microstrips or slight deviations from the doping design values. Similar observations on $\Delta V_{op}$ have been made for 100 μm thick and 300 μm thick sensors.

It can be observed in Figure 18 that smaller (deep) p-wells result in increased $V_{dpl}$ and $V_{pt}$. This can be interpreted as follows. Large p-doped surfaces below the (deep) p-wells create wider pn-junctions with the n-doped epitaxial layer, thus facilitating the depletion of the underlying epitaxial layer at lower voltages. On the other hand, large (deep) p-wells also lower the potential barrier that prevents the direct flow of holes towards the substrate. This results in the earlier onset of the punch through hole current between the (deep) p-wells and the backside p+ region. Sensors without the deep p-well showed higher reference voltages. In fact, the presence of a deep p-well reduces the epitaxial layer thickness below the p-wells, thus requiring a lower voltage to achieve both full depletion and the onset of punch-through currents.

For sensors with too large deep p-well, a phenomenon that we defined as *channel choking* was observed. It consists in the closure of the conductive channel below the collection n-well due to the lateral merging of the closely adjacent depletion regions formed at the junctions between the deep p-wells and the n-epitaxial layer. In this condition, even though the space charge region of the backside junction has not reached the surface yet, the n-wells are already isolated from one another at Vback = 0 V. Hence, the process of charge collection is inhibited by the strong potential barrier present below the n-wells. No channel choking was observed for sensor layouts without the deep p-well.

With a minimum-sized collection n-well and within the foundry’s process requirements, a maximum gap of about 2.1 μm is allowed to have enough space for monolithically integrated CMOS electronics in the inter-strip region. This specific layout option is not affected by the channel choking and is, hence, operational. If, however, NMOS-only electronics is integrated along the strips, the smallest p-well size considered in our study can be used.

**Figure 19:**
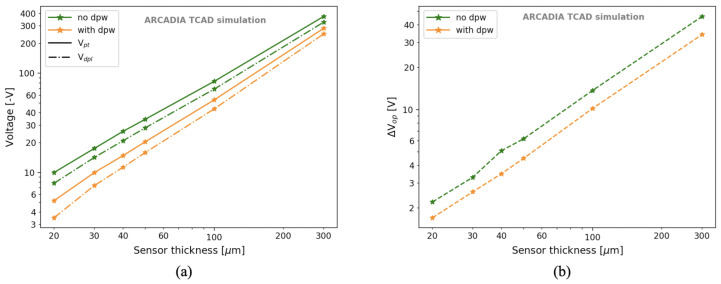
Dependence of $V_{dpl}$ and $V_{pt}$ (**a**) and of the operating voltage range $\Delta V_{op}$ (**b**) on the sensor thickness.

For completeness, Figure 19a illustrates the dependence of $V_{pt}$ and $V_{dpl}$ on the sensor thickness for the sensor layout with minimum sizes for the n-well and for the (deep) p-well. The trend is linear over a wide range of thicknesses, both with and without the deep p-well. In addition, the operating voltage $\Delta V_{op}=V_{pt}-V_{dpl}$ linearly increases with the sensor thickness, as shown in Figure 19b. The sensor thickness investigated was extended down to 20 μm, well below the smallest thickness (i.e., 50 μm) of the sensors that will be produced in the first ARCADIA engineering run, as the study of thin sensors was functional for improving the sensor timing performance in the simulations.

The voltage $V_{pd}$ at which the power density is 0.1 mW/cm${}^{2}$ was found to be about 4–5 V above $V_{pt}$ for 50 μm thick microstrips, 7–8 V for 100 μm thick microstrips, and 18–20 V for 300 μm thick microstrips when the deep p-well was included.

**Figure 20:**
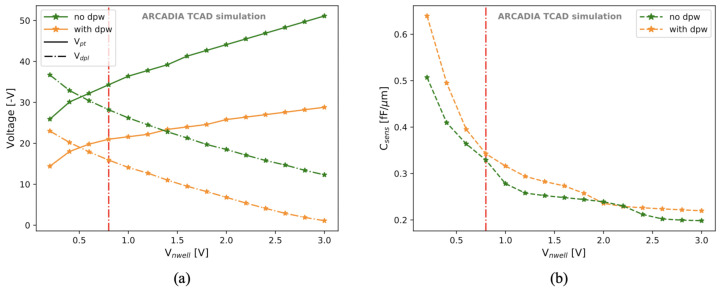
$V_{dpl}$ and $V_{pt}$ (**a**) and sensor capacitance (**b**) as a function of $V_{nwell}$. The vertical red line indicates the default value of $V_{nwell}=0.8$ V.

The $V_{nwell}$ voltage was varied with the aim of finding possible improvements in the sensor performances. The results for $V_{dpl}$ and $V_{pt}$ are shown in Figure 20a, where the vertical red line indicates the default value of 0.8 V. A minimum $V_{nwell}$ of about 0.5 V is necessary to satisfy the condition $|V_{pt}\left|>\right|V_{dpl}|$. Moreover, an increase in $V_{nwell}$ has several interesting effects. First of all, it allows the sensor full depletion to be reached at lower (in absolute value) backside voltages. Secondly, it also shifts the onset of the punch through towards larger $|V_{back}|$, thus increasing the operating range $\Delta V_{op}$. Finally, as shown in Figure 20b, higher $V_{nwell}$ implies lower sensor capacitance. At $V_{nwell}=3$ V, the capacitance can be reduced to about 0.2 fF/μm.

### 3.4. Charge Collection Studies

**Figure 21:**
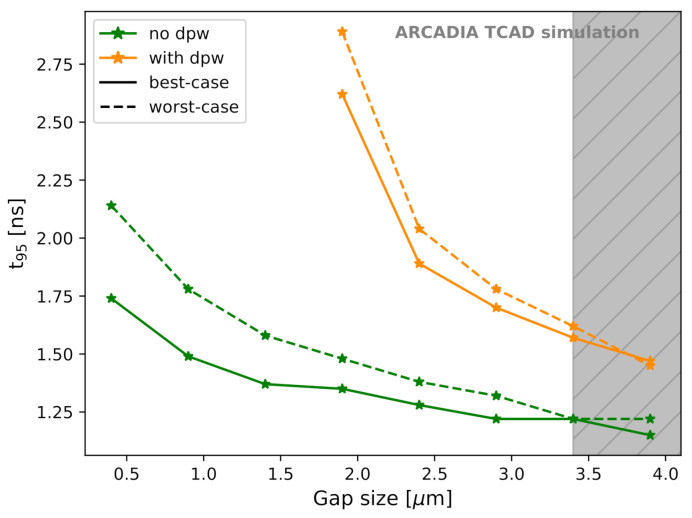
$t_{95}$ as a function of the gap size for best-case and worst-case scenarios.

As described in Section 2.2.6, TCAD transient simulations were used to study the charge collection dynamics. In order to select the layouts with the optimal performance in terms of fast and uniform charge collection, the effect of the (deep) p-well size on the charge collection time at $V_{back}=V_{pt}$ was evaluated. The time $t_{95}$ needed to collect 95% of the total charge deposited in the simulated sensor domain is plotted in Figure 21 for 50 μm thick sensors and LET = $1.28\times 10^{-5}$ pC/μm (1 MIP) as a function of the gap size and with fixed minimum n-well size.

Microstrips with large gaps, and hence small (deep) p-wells, are to be preferred for fast charge collection. The reason for this is a higher $|V_{pt}|$, which enables sensor operation at a larger $|V_{back}|$. The consequent stronger electric field in the sensor results in higher charge velocity in the silicon substrate. For the same reason, microstrip sensors without deep p-well revealed a significantly faster charge collection in both the best-case and the worst-case scenario. The options with small (deep) p-wells also show uniform charge collection for different particle incidence positions. The difference in $t_{95}$ for the best-case and worst-case scenarios is below 0.1 ns for the fastest permitted options. This result is also achieved, thanks to the fine microstrip pitch of 10 μm. The channel choking, as described in Section 3.3, limits the deep p-well size as the potential barrier below the sensing node slows down the electron collection. This problem, as shown in Figure 21, can be avoided by removing the deep p-well.

A gap size of about 2.1 μm, which guarantees enough space for embedded CMOS electronics in the inter-strip region according to the foundry’s layout rules, is not in the channel choking region. Hence, it preserves the sensor functionality without a dramatic slow down of the charge collection process.

**Figure 22:**
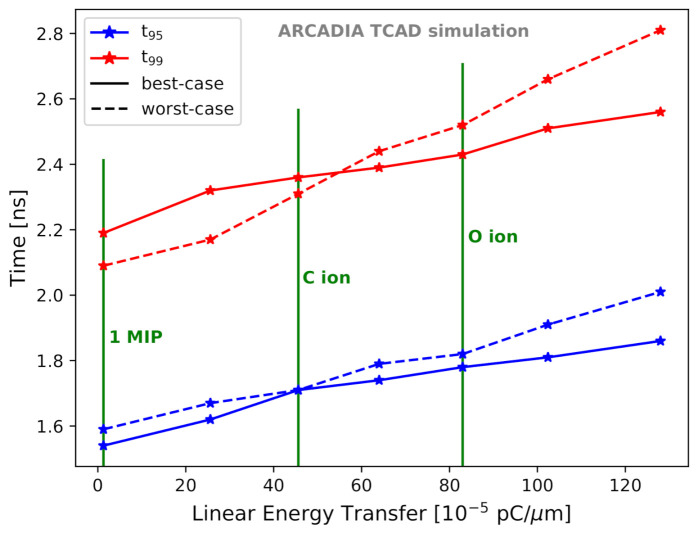
$t_{95}$ (blue) and $t_{99}$ (red) as a function of the LET for best-case and worst-case scenarios.

Figure 22 demonstrates that the proposed MAMS guarantee fast sensor response also under heavily ionizing particles. The charge collection time is only weakly proportional to the charge deposited by the incident particle within an LET range of 1.28 × 10${}^{-5}$ pC/μm to 128 × 10${}^{-5}$ pC/μm. A 50 μm thick sensor was considered in Figure 22, and the LET values corresponding to 1 MIP, carbon (C) ion, and oxygen (O) ion at their minimum of ionization are highlighted as vertical green lines. $t_{99}$ is added to show that the time needed for complete charge collection is only slightly larger than $t_{95}$, due to a small fraction of charge collected by the strips adjacent to the central one. $t_{95}$ and $t_{99}$ were never found to exceed 2 ns and 3 ns, respectively, in 50 μm thick sensors.

As we discussed in Section 3.4, the first strategy for improving the timing performance of the proposed microstrip sensors is to remove the deep p-well in order to obtain higher $|V_{pt}|$. However, we also investigated other ways to increase $|V_{pt}|$ and to speed up the charge collection. In particular, as shown in Section 3.3, a larger $V_{nwell}$ shifts the onset of the punch-through current towards higher $|V_{back}|$. Hence, we explored the effects of $V_{nwell}$ on the charge collection time.

In a strip readout system, timing information can be retrieved only from the strips collecting most of the charge (i.e., strip(s) number 1, following the nomenclature of Figure 8), as they provide a signal with sufficiently large SNR. Therefore, in order to study the sensor timing performance and after verifying through $t_{95}$ that the total deposited charge is quickly collected in the whole simulation domain, we considered the time $t_{95}^{central}$ needed to collect 95% of the charge in the central strip(s).

**Figure 23:**
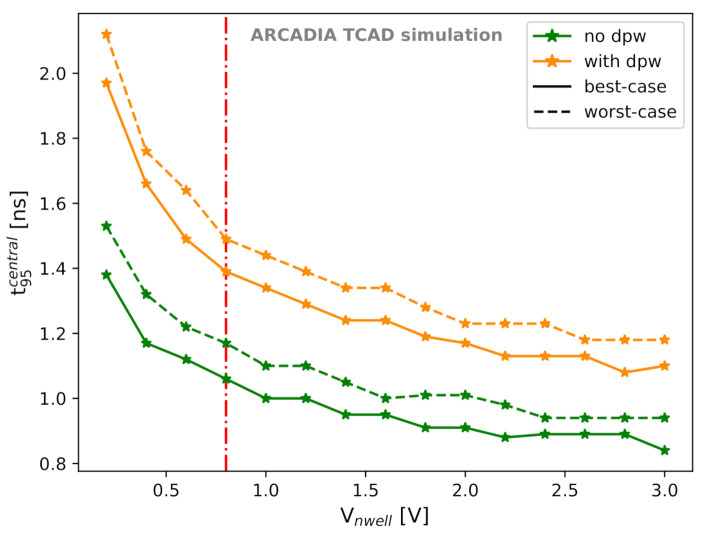
$t_{95}^{central}$ as a function of the voltage $V_{nwell}$ applied to the sensing node for best-case and worst-case scenarios. The vertical red line indicates the default value of $V_{nwell}=0.8$ V.

Figure 23 shows the dependence of $t_{95}^{central}$ at $V_{back}=V_{pt}$ and with LET = $1.28\times 10^{-5}$ pC/μm on the voltage applied to the sensing node. A 50 μm thick sensor with a layout optimized for fast charge collection was considered. A significant improvement could be reached at larger $V_{nwell}$. For the option without deep p-well and at $V_{nwell}=3$ V, $t_{95}^{central}$ is 0.84 ns in the best-case and 0.94 ns in the worst-case scenario. If we assume an electron drift saturation velocity of $∼1\times 10^{7}$ cm/s in silicon at a temperature of 300 K [47], the minimum drift time for electrons that have to cover a 50 μm distance is 0.5 ns. This explains the saturation observed in Figure 23 and demonstrates the fast charge collection and the promising timing capabilities of the proposed MAMS.

**Figure 24:**
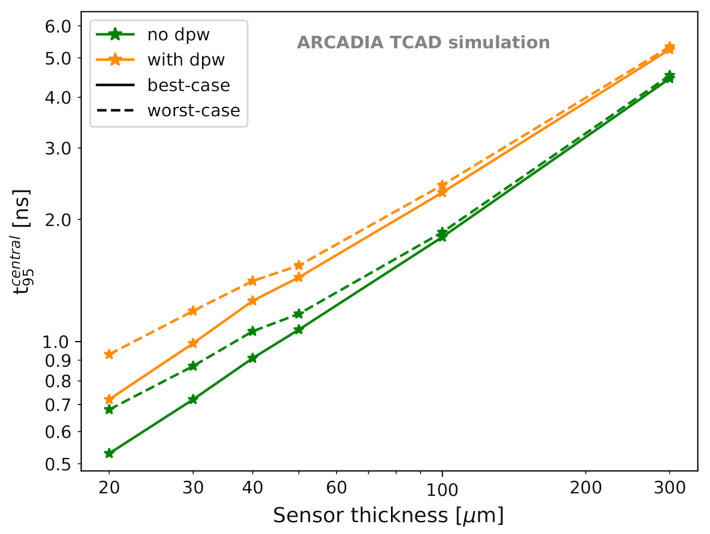
$t_{95}^{central}$ as a function of the sensor thickness for best-case and worst-case scenarios.

A way to further reduce the collection time is to explore thinner sensors. Figure 24 demonstrates that the charge collection time $t_{95}^{central}$ is proportional to the sensor thickness. For these simulations, $V_{nwell}$ was set to 0.8 V and a 1 MIP LET was considered. Even at thicknesses as large as 300 μm, $t_{95}^{central}$ does not exceed 6 ns. In the best-case scenario without the deep p-well, reducing the sensor thickness from 50 μm to 40 μm, 30 μm, and 20 μm results in a decrease in $t_{95}^{central}$ of 15%, 33%, and 50%, respectively. Analogous proportionality was observed for $t_{95}$. Therefore, for future production runs, thinner sensors could be considered for the enhancement of the timing performance.

A set of TCAD simulations was dedicated to study the charge sharing among adjacent microstrips when particles with different LETs traverse the sensor. Charge sharing is relevant for improving the spatial resolution, especially with analog readout, and is enhanced by fine microstrip pitches and large sensor thicknesses. On the contrary, it is reduced at higher $V_{back}$ for a fixed sensor thickness.

**Figure 25:**
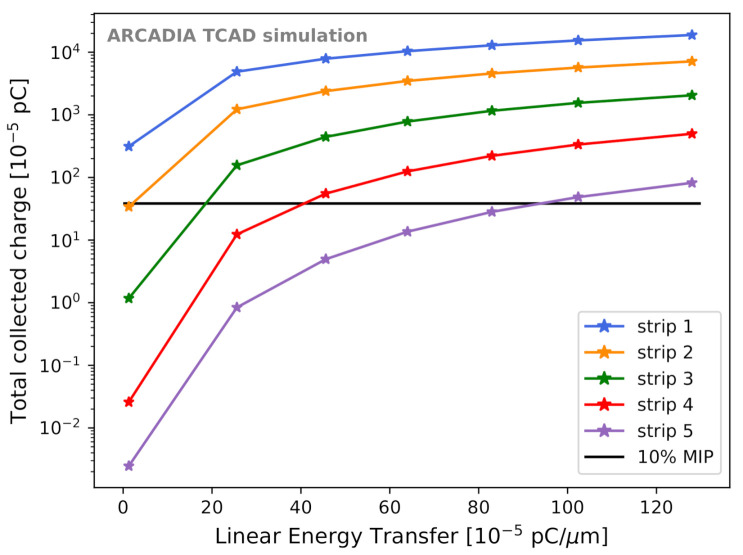
Charge sharing among adjacent microstrips. The total charge collected by strips 1 to 5 (following the nomenclature illustrated in Figure 8) is shown as a function of the LET.

In Figure 25, the case of a 300 μm thick sensor at $V_{back}=V_{pt}$ is presented for the best-case scenario. The total charge collected by each strip (identified using the nomenclature of Figure 8) is plotted versus the LET. The black horizontal line indicates a possible charge threshold corresponding to 10% of a MIP at the single strip level. A comparison with the sensors that will be produced in the first ARCADIA engineering run will allow deeper investigation on the charge sharing, a fine tuning of the simulations and studies aimed at evaluating the spatial resolution of 10 μm pitch MAMS.

## 4. Conclusions

In this work, we presented detailed TCAD simulations of CMOS-based FD-MAMS, which may find use for tracking and timing in particle and nuclear physics, space and medical applications. The results of the TCAD simulations, performed to design the 10 μm pitch FD-MAMS, demonstrate their fast and uniform charge collection, which encourages their practicality for various applications, even under heavily ionizing particles. The effect of surface ionizing radiation damage was investigated, and the layout parameters were varied to achieve a minimum capacitance, beneficial for electronic noise reduction. The possibility to operate the sensor in full depletion and at low-power density (i.e., before the onset of the punch through current) was verified in the simulations. A preference for small collection diodes and small (deep) p-wells emerged for obtaining lower capacitance and faster sensor response. Additionally, even with a pitch of only 10 μm, it was found that there is enough space for hosting transistors in the inter strip region according to the foundry layout rules without slowing down the charge collection process. The first FD-MAMS samples will be produced in the upcoming ARCADIA engineering production run in mid 2021 and will allow the simulation results to be compared with experimental data from electrical characterization, laser and beam irradiation tests. The promising results of the FD-MAMS simulations will translate into further R&D activities to enhance the sensor performance in terms of low capacitance and high timing and spatial resolution.

## Figures and Tables

**Figure A1:**
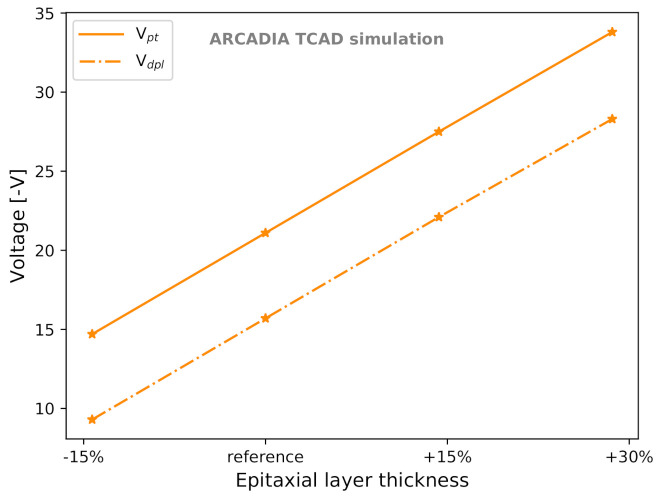
$V_{dpl}$ and $V_{pt}$ as a function of the epitaxial layer thickness, expressed as percentage variation with respect to the reference thickness.

## Data Availability

Not applicable.

## Appendix A. Expected Effects from Epitaxial Layer Thicknesses

Possible variations in the epitaxial layer thickness of (−15%; +30%) communicated by the foundry with respect to the reference value induced us to investigate their effect on the operating parameters. While the sensor capacitance was observed not to be influenced, both $V_{dpl}$ and $V_{pt}$ showed a linear dependence on the epitaxial layer thickness. This behavior is presented in Figure A1.
